# In the family: access to, and communication of, familial information in clinical practice

**DOI:** 10.1007/s00439-021-02401-0

**Published:** 2021-12-08

**Authors:** Anneke Lucassen, Angus Clarke

**Affiliations:** 1 Clinical Ethics and Law, Faculty of Medicine, Southampton University, Southampton, UK; 2 Medical Genetics, Cardiff, UK; 3 Centre for Personalised Medicine, Wellcome Centre for Human Genetics, Nuffield Department of Medicine, University of Oxford, Oxford, UK

## Abstract

How an individual’s genetic information is governed by confidentiality, and how the interests of others—such as close relatives—in knowing such information might be respected, has been the topic of much debate ever since genetic testing has become more prevalent. In this paper, two authors who often appear to have different views on familial disclosure, discuss where they agree on this topic.

How an individual’s genetic information is governed by confidentiality, and how the interests of others—such as close relatives—in knowing such information might be respected, has been the topic of much debate as genetic testing in healthcare has become more prevalent. With others, AL and AC have for 20 years run a multidisciplinary forum in the UK that discusses issues raised by particular cases that are exercising the health professionals involved. AC and AL often appear to have different views in such cases. In a BMJ debate (Lucassen and Clarke 2007), AC argued that a person’s genetic status is usually very much their personal information, and thus, it is also very much up to them how they share this with relatives, who may [or may not] be at risk of a similar inheritance. AL on the other hand considers genetic information as [potentially] familial, so that it could be shared with relatives who might be at risk. Such sharing, she argues, can often be separated from the duties of confidence that are clearly owed regarding clinical information. They are agreed, however, that information generated by the genetics diagnostic laboratory in one individual should be available for use by the laboratory to test at-risk family members without the first individual being able to veto such use. Such familial information can be very important in targeting the genetic test for the relative, significantly improving the positive and negative predictive values of the genetic test in the relatives. AL maintains that the relevant (pathogenic) genetic laboratory finding is familial information, unlike the personal information about how the individual is affected by the relevant disorder. The genetic information is therefore not personal in the same way, so that its application for the benefit of others does not amount to a breach of confidence. AC agrees that the information needs to be applied to the benefit of at-risk relatives, but disagrees about the justification for this. He argues both that the technical, molecular information ‘belongs’ to the laboratory and health service more than to the index case or ‘the family’, and that any right the individual has to control over this information cannot override the health service’s duty of care to the relatives. If relatives need to be alerted, the extent of any confidence breach must be kept to a minimum. In practice, therefore, AC and AL may agree about what needs to happen, but will sometimes differ in their reasons why.

There is of course a tension between respect for one person’s confidence and the need for others to be given information that may be important for their health. To treat such information as available for use on behalf of other family members, even when it has been generated by testing one particular individual, may help to resolve this tension in practice but care should be taken not to divulge personal, clinical information in this process. In this discussion paper, we aim to develop a better understanding of the normative values that flow from the discovery of potentially familial information in one person. We start with a clinical scenario.

## Scenario A: FAP

John has familial adenomatous polyposis (FAP) confirmed through mutation testing. Whilst this might be a de novo mutation, and so the relevance for siblings and parents is not yet clear, for John’s three children (aged 10, 13, and 15 years), each has a 50% chance that they have inherited the condition and would then benefit from regular gastro-intestinal surveillance for polyps and tumours, followed by surgical removal of the colon when polyp burden becomes high. Such a programme will often be life-saving and this may also apply to John’s siblings and one of his parents.

Part of the standard care of an individual diagnosed with FAP will be to discuss who else in the family needs to know this information. Usually, such information is passed to others, perhaps with the help of a general letter from the health professional outlining what a relative needs to do to avail themselves of testing. The information to be made available is (1) the diagnosis (the name of the condition for which the relative is at risk), and (2) its potential implications, including the importance of appropriate medical interventions. In addition, (3) it is important for any genetics laboratory testing the relatives to have access to the molecular information about John’s *APC* gene mutation, in order that they can target testing appropriately given that different families will have different mutations (variants) within this gene.

When family’s channels of communication function smoothly, those at risk can be offered monitoring in good time. However, such channels may be disrupted by poor relationships, geographical distance or for a variety of other reasons such as not wanting to be the bearer of bad news, not knowing when is a good opportunity to share unwelcome information, or a lack of concern about a particular relative (Forrest et al. 2003; Featherstone et al. 2006). The recipient of the information may be grateful, but they might instead misunderstand or reject the information and thus not seek the testing that is indicated.

## What should a clinician do when they come to realise that a relative at risk has not been offered the information?

AC and AL are agreed that a first step is to promote the passing of information within the family, perhaps by asking other family members to be involved or by highlighting what is at stake through a failure to communicate. At this point in the debate, questions often arise such as “whose duty is it to alert relatives of their risk?” We consider that, if such a duty exists, it is primarily the responsibility of the index patient, such as John, but that the health professionals may also have a role to play. Most often, John, or patients like him, are charged with such communication and health professionals have no means of checking whether it has really taken place. Relatives—if they do get to hear—might be referred to different clinical services in other parts of the country, so there would be no direct way of knowing whether they had been appropriately informed or tested: it is only when a clinician comes to hear of non-communication that they may need to consider whether or how to bypass the block. Here, AC has a tendency to focus on the difficulty of appearing to breach John’s confidence by alerting his relatives. In contrast, AL has a tendency to focus on alerting relatives of their risk without any direct reference to clinical information about John. Both consider that contacting a relative’s GP—for example—can be a helpful way to ensure that the appropriate information is passed on. AC considers that this will often be difficult to do without that relative then inferring something confidential both about John’s health and also about John’s reluctance to pass on the information. AL, however, thinks that general information, such as “you may have inherited a tendency to bowel cancer”, will not inevitably point to John or the fact that he has withheld or found it difficult to communicate this information. Inferences about where such an ‘inherited tendency’ has come from may of course be made, and might lead to awkward family discussions with John, but this may be necessary to accept as a cost of resolving the tension between privacy and disclosure that family members make. Furthermore, AL considers that any inferences made do not mean that the health professional has breached any confidences, since people make inferences about inheritances all the time, and often independently of health professional input.

It may of course be difficult in practice to contact relatives via their GP, if the name or date of birth of the relative is not known. In that case, even the efficient system of NHS patient tracing within the UK will not be able to identify a GP to contact. It is where such information is known that we differ in our approach in some circumstances.

Several factors will shape the strength of the case to pursue disclosure to relatives, such as the immediacy and the degree of risk to others in the family and the extent to which interventions can mitigate this. Where the risk is to the physical health and even survival of a family member, and medical intervention is known to provide an effective remedy, the case for insisting on, or even forcing, such disclosure is stronger. In John’s case, we would both make efforts to identify the estranged younger brother, Thomas, whose existence John told us about during construction of his family pedigree but with whom he has had little contact over the past decade because of an unpleasant family row. We are agreed that Thomas is at risk and that bowel screening may help save his life if he too has inherited this condition. We are also agreed that the strength of any obligation we feel to disclose will depend on the disorder in question, the proximity of relatedness of the relative (and thus the risk they are at), and the available interventions.

## Scenario B: The ABC versus St George’s case

A recent genetics case that came to the English courts highlights some of our differences in disclosure practices. This case concerned the condition Huntington’s disease (HD), a genetic condition with the same mode of inheritance as FAP but no established, evidence-based intervention to ameliorate the course of the condition; the only means of prevention is through reproductive choices, although clinical trials of early treatments may well change this over the next few decades.

This case was a claim of negligence by a woman against the professionals caring for her father. The professionals suspected, and later knew, that her father had HD, but, despite their concern for her (and her sister), considered that they could not tell the sisters of their own risk without their patient’s consent, which he forcefully denied. This view persisted even when they learned the woman was pregnant, although there was considerable disagreement within the psychiatric team looking after the man. Whilst the court case then focused on whether the non-disclosure was negligent, we are concerned here with whether disclosure might have been appropriate. The court ruling highlighted that, in such cases, health professionals are expected to weigh up the harms of breaching the father’s confidentiality with the harms of non-disclosure to relatives. Whilst this might be a difficult balancing act, professionals would be required to act on the outcome of their balancing and that the patient’s consent (or lack of it) could not be the trump card that the psychiatric team considered it to be. It was acknowledged by the judge that the court’s decision might well have been different if there was an established medical intervention able to prevent or treat HD.

It may be noted that the framing of the judgement suggests that the only factors to be weighed in coming to a decision are the likely consequences of the different courses of action. Whilst agreeing with the judgement, AC is not satisfied that this is all there is to be considered. Professionals have obligations they need to respect that are not simply the outcome of a utilitarian calculus Whether or not ABC would have terminated her pregnancy cannot now be known, but it is the denial of her choice in the matter that was at stake. AL agrees with the need to balance competing interests, and act on that decision, but disagrees that such a balancing act was in fact considered possible by the psychiatric team in the ABC case. Evidence given in court suggested that the psychiatric team wanted to share the information but considered the law prevented them from doing so without the father’s consent, and therefore, the judgement was helpful in highlighting that this interpretation was wrong.

Whilst this case digresses from our discussion somewhat, as it focuses on whether the non-disclosure arose from negligence. Instead, we are concerned with when it might be appropriate for health professionals to disclose information discovered in one person to their relatives without their consent and even against their wishes. However, it is helpful in that it highlights some of the differences in our views. We agree that disclosure to his daughter by the patient, or with his permission, would be the preferred path. However, AC considers that the responsibility for disclosure to the daughter rests with the patient for as long as he retains the capacity to do so and that the health professionals have an obligation not to breach his confidence. The shame or social stigma of a diagnosis of HD, the lack of available intervention, and the fact that the daughter would likely have concluded that her risk of HD must have come from her father are additional factors that AC considers weigh against disclosure. AL considers that the health professionals involved could have alerted the daughter to her risks (rather than her father’s diagnosis) and that this could have been done without shame or social stigma coming to the fore any more than had already been raised for this family. Furthermore, the diagnosis would be sooner or later have become apparent to the daughter and then, when she learned of her own risks, she might legitimately have asked why this information had been withheld from her, and feel aggrieved that it had been.

For AC, the fact of the daughter’s pregnancy does not add to a disclosure imperative, given that the large majority of women carrying a fetus at risk of HD seek neither prenatal diagnosis nor termination of the pregnancy. AL considers that the highly unusual set of circumstances of the ABC case are not helped by the decisions of the large majority in other pregnancies. She considers that being unable to exercise reproductive autonomy is akin to not being offered a medical intervention to influence the course of a disease.

With a different disease, such as FAP, where the ignorance of relatives would have denied them appropriate medical care, AC would have pushed harder for the patient to make a disclosure and would then have disclosed himself if the patient had insisted on not doing so. AL, whilst she agrees with these points, considers that the communication surrounding heritable risks can be handled differently: in the ABC case, had the professionals involved separated the clinical diagnosis from the genetic one, they could have called upon factors already known to the daughter—the symptoms she could see in her father, her family history of these symptoms—to hint at a possible inherited condition: “it is possible that his symptoms might be explained by an heritable factor; if you would like to know more, we can refer you to a genetics service”. This needs not to have involved any breach of confidence at all and could have alerted her to information that she—in retrospect—would have wanted, and which in any case could not have been kept a secret forever.

We are agreed that this nuanced disclosure might well have resolved the issues.

## Harms that may result from non-communication

In the context of FAP, what is at stake for individuals who are at risk of disease but unaware of their risk? They are at risk of an early onset of malignancy, that may be slow to be diagnosed (and therefore fatal) because of the unusually early age of onset of the bowel cancer in this condition, which may not present with physical symptoms until it is quite advanced.

In the ABC case, which concerned HD, there was no intervention or treatment known to prevent the condition, and so the urgency for disclosure was on the face of it somewhat different. However, the daughter in the ABC case argued that she would have had a termination of pregnancy if she had known that she herself had inherited HD and might therefore become unable to raise her child to adulthood. AL considers this a form of intervention that might have justified disclosure in the same way as treatment might, whilst AC considers there to be a different quality of obligation to disclose in this setting. The judge in the ABC case was not convinced that the daughter would have had a termination—despite her evidence—thus suggesting that the judge too considered this a different type of intervention to bowel surgery to reduce the risk of cancer.

## Genetic test results

The information being communicated within a family is usually about a genetic condition. For example, “I have Huntington’s disease” or “Testing has shown that my breast cancer is an inherited form”. For the relative to be tested, the laboratory process is much simpler and more reliable if the precise pathogenic genetic variant (“mutation”) responsible for the family’s condition is known. Should this technical, laboratory-generated information be treated as of the same status as the clinical information about individual patients? We realised that here might lie a solution to resolve at least some of our differences.

Whilst such results are usually given the same status as clinical information about particular patients, because a person’s name and other identifying details will be on the report, we wondered whether a system might be devised where that technical report was made available to members of a family without all of the identifying details: “Members of family X can seek advice about genetic testing with this anonymised result to guide their care”. How we define families, and which members are indeed at risk, might lead to problems with this approach—someone might need to know how a relative is related to the proband to know whether testing is indicated—but we imagine that processes could be defined to help resolve such concerns.

It should also be remembered that the testing of many genes is much simpler and more rapid than in the past, so that the imperative to know the details of the proband’s genetic test result has softened. It would be possible either to test all the genes in which a mutation is known to be a likely cause for the condition or even to perform whole-genome sequencing, but, until such testing is available to all, one would still need to find a means to alert a relative to their interests in having such a test. The requirement for knowledge of the particular family mutation might weaken, but such solutions would require clinicians to fudge their explanations for testing in some way and the more targeted, rapid and cheaper test, that knowledge of the particular mutation allows, would be denied to at-risk individuals.

Perhaps, the current COVID pandemic has also helped us to come to this suggested approach: to access testing, it is not necessary to know ***whom*** the contact is that one has been exposed to, but one might know very well who has put us at risk. Notwithstanding the significant differences between horizontal transmission of an infectious agent and the vertical transmission of genetic information, there are similarities in that the test results are to be used to help inform others rather than breach clinical confidences.

## Consent to use test results

When the possibility of a particular condition has been conveyed to an at-risk relative, who then seeks genetic advice to clarify their risk status, we both agree that the genetic laboratories involved with the family be permitted to utilise relevant test results without the need for specific approval by each individual, so that the best advice can be given to relatives at risk with the minimum of inconvenience and delay. We go further and suggest that access to DNA samples (stored routinely for years after genetic testing) should also be available for familial testing, since a positive control sample can improve the sensitivity of a test. Consent from an individual is crucial for their testing, but not we argue for subsequent use as a quality control for the testing of relatives. We would welcome a discussion on how this might be done without attaching the name of the individual tested to this disclosure.

## Separating clinical and genetic information

Our favoured approach would be to separate the diagnostic label and its clinical implications from the molecular basis of that diagnosis. Thus, genetic results—or indeed the stored DNA samples—should be accessible to the laboratory and the healthcare system without specific consent, if it is to be used to benefit other family members who are also users of the healthcare system. This would make no difference to the need to maintain respect for the confidentiality of clinical information about the index case, but would ensure that the molecular results on one family member could be used to test their relatives, if any request such tests. Questions about the ‘ownership’ of genetic information in this context may function as a distraction; we prefer to say that the laboratory has a right of access to the information and/or the sample, or has the right to make use of it, rather than entering into a discussion of what ‘ownership’ or ‘belonging’ means in this context. Indeed, as the laboratory generated the information in the first place, a refusal by a patient to allow the laboratory to make use of the information for the benefit of others amounts to the perverse imposition of an ‘obligation to forget’. Referring to a right of access to the information or the right to make use of it is to be preferred (Ballantyne 2020; Liddell et al. 2021). If we use the term ‘belonging’ at all, it is because it so often comes up in discussions related to these issues.

In the context of a national healthcare system, there is a discussion to be had about how widely the term, ‘the laboratory’, can be taken to mean. We would not want to focus this to the specific laboratory that identified the mutation in the family’s index case. We would certainly want an NHS laboratory in Cardiff (Wales) for example, to be able to make use of information generated by the corresponding laboratory in Salisbury (England). We would hope that the term ‘laboratory’ would be taken to cover at the very least all laboratories within the UK’s four National Health Services; any other approach could result in endless wrangling as to the administrative boundaries between different laboratory regions. At present, that is not how laboratories function within our National Health Services. The need to produce documentation of consent for information to be shared is frequently an impediment to good practice, when no breach of clinical information would in fact result from the use within the laboratory of information about one individual in testing another.

Although we do not have the space to develop this fully, there are clear parallels between this point and the need to compare clinical and molecular findings in population-based databases if we are to improve our interpretation of molecular genetic variants that are encountered in diagnostic genetic tests. In a sense, the entire species of *Homo sapiens* needs to be regarded as a single, extended family if we are to be able to benefit more fully from the advances in genetics. Safeguards need to be available, as it would not be acceptable to force the inclusion of unlimited phenotypic data about individuals in internationally accessible databases whose security could never be guaranteed. However, there perhaps needs to be a recognition of a certain *quid pro quo* when accessing genetic laboratory services. If we seek to benefit from genetic testing, should we be required to make at least some, de-identified information available to assist with the interpretation of genetic tests of others in the future?

## Conclusion

Different perspectives exist about the status of genetic information concerning one individual, that may be important and relevant to the healthcare of other family members. We wish to side-step many of the unproductive arguments in this area by recognising that health services should be able to make use of technical information generated within the healthcare system for the benefit of not only the individual patient but also their family members. In other words, to draw upon technical information already known does not amount to a breach of confidence. Returning to our FAP case, we are agreed that our duty as John’s clinician includes considering how his close relatives may be alerted of their risks and offered interventions, and that if John does not do so, we need to make some attempts to facilitate communication. Indeed, genetic services have long operated with the perspective that their ‘patient’ is not solely the individual in front of them in clinic but is—potentially—the extended family (Parker and Lucassen 2003).

We wish to restrict the term ‘breach of confidence’ to the passing of clinical information about one family member to another by a health professional without consent; and so to limit the tendency to broaden the scope of this term to include any application of information held about one person by the healthcare system to the benefit of another family member even when no clinical details are shared. We should also remember that patients often assume that their health professionals would share information about them with family members as clinically appropriate. Dheensa et al (2016) showed that, in contrast, health professionals thought that their patients would not want them to do this, or that the law prohibited them from doing so; indeed professionals can overemphasise these obstacles to good clinical practice.

The parallels with information about exposure to COVID may be a topical point of comparison. With state-endorsed COVID apps, one can be given information that one has been close to an individual later found to have been infected, without any breach of confidence as to who the person was or any further details about them, although those additional items of information are of course held within the system. We may make inferences about who has infected us, but these inferences may well be wrong, just as inferences about inheritances might be.
